# The Promotion of a Bright Future and the Prevention of a Dark Future: Time Anchored Incitements in News Articles and Facebook’s Status Updates

**DOI:** 10.3389/fpsyg.2018.01623

**Published:** 2018-09-13

**Authors:** Danilo Garcia, Karl Drejing, Clara Amato, Michal Kosinski, Sverker Sikström

**Affiliations:** ^1^Blekinge Center of Competence, Blekinge County Council, Karlskrona, Sweden; ^2^Department of Psychology, University of Gothenburg, Gothenburg, Sweden; ^3^Network for Empowerment and Well-Being, Gothenburg, Sweden; ^4^Stanford Graduate School of Business, Stanford University, Stanford, CA, United States; ^5^Department of Psychology, Lund University, Lund, Sweden

**Keywords:** future, latent semantic analysis, past, present, prevention focus, promotion focus, time-anchored incitements

## Abstract

**Background:** Research suggests that humans have the tendency to increase the valence of events when these are imagined to happen in the future, but to decrease the valence when the same events are imagined to happen in the past. This line of research, however, has mostly been conducted by asking participants to value imagined, yet probable, events. Our aim was to re-examine this time-valence asymmetry using real-life data: a Reuter’s news and a Facebook status updates corpus.

**Method:** We organized the Reuter news (120,000,000 words) and the Facebook status updates data (41,056,346 words) into contexts grouped in chronological order (i.e., past, present, and future) using verbs and years as time markers. These contexts were used to estimate the valence of each article and status update, respectively, in relation to the time markers using natural language processing tools (i.e., the Latent Semantic Analysis algorithm).

**Results:** Our results using verbs, in both text corpus, showed that valence for the future was significantly higher compared to the past (future > past). Similarly, in the Reuter year condition, valence increased approximately linear from 1994 to 1999 for texts written 1996–1997. In the Facebook year condition, the valence of the future was also significantly higher than past valence.

**Conclusion:** Generally, the analyses of the Reuters data indicated that the past is devaluated relative to both the present and the future, while the analyses of the Facebook data indicated that both the past and the present are devaluated against the future. On this basis, we suggest that people strive to communicate the promotion of a bright future and the prevention of a dark future, which in turn leads to a temporal-valence asymmetrical phenomenon (valence = past < present < future).

***“****I have a dream that one day this nation will rise up and live out the true meaning of its creed: “We hold these truths to be self-evident, that all men are created equal.”*

I have a dream that one day on the red hills of Georgia, the sons of former slaves and the sons of former slave owners will be able to sit down together at the table of brotherhood.

I have a dream that one day even the state of Mississippi, a state sweltering with the heat of injustice, sweltering with the heat of oppression, will be transformed into an oasis of freedom and justice.

I have a dream that my four little children will one day live in a nation where they will not be judged by the color of their skin but by the content of their character.

I have a dream today!”

Martin Luther King, Jr., 28th of August 1963, at the Lincoln Memorial, Washington, DC, United States

## Introduction

A myriad of theories and empirical studies illuminate our understanding of how we evaluate the past, the present, and the future (e.g., Higgins, 1997; Trope and Liberman, 2000; Caruso et al., 2008; Kurtz, 2008). This line of research has mostly been conducted by asking participants, in experimental conditions, to value imagined positive and/or negative events as occurring either back or forward in time (for some exceptions see: Wilson et al., 2012). At a general level, people usually assign higher values to future events compared to past events (e.g., Trope and Liberman, 2000; D’Argembeau and Van der Linden, 2004; Van Boven and Ashworth, 2007; Caruso, 2010). In the present study, we use data from a Reuter’s news corpus and Facebook status updates or “real-life data” (i.e., peoples’ actual narratives about past, present, and future events). These real-life data contain statements of both positive and negative events with frequencies that more closely reflect their occurrence in peoples’ life, in contrast to controlled experiments with either positive or negative events in equal numbers. We measured the valence^1^ of the statements using a semantic statistical method, namely, the Latent Semantic Analysis (LSA; Landauer et al., 1998) algorithm. Thus, the present study makes an important addition to the existing literature because it is based on ecological data from multiple events over time, that we organized in statements of events remembered or imagined to happen in the past, the present, and the future. In sum, we use a larger sample, actual behavior, and data with natural validity that circumvent the limitations of self-reports.

Most experiments on how humans evaluate events in different temporal dimensions ask participants to imagine fictional, yet probable, scenarios. For example, participants are asked to imagine performing a mundane task (e.g., entering data into a computer) and then to rate, at random, the amount of money they would like to get paid if they will perform the task in the future versus if they had already performed the task in the past. Intuitively, one might suspect small differences, however, participants who imagine doing a mundane 5-h task one month in the future demand twice as much more money compared to participants who imagine having completed the same task one month ago (Caruso et al., 2008). This temporal asymmetry is stable across various types of judgments, such as, monetary gain, generosity, and pleasure (e.g., Caruso et al., 2008). In addition, moral transgressions are judged more negatively and deserving more punishment if people imagine them to happen in the future rather than if these transgressions already have happened in the past (Caruso, 2010). In other words, this line of research suggests that when we create a representation of an event happening in the future, both positive and negative events seem to increase in their evaluative magnitude, but to decrease when we imagine that the same events have already happened in the past. One possible reason for this is that people see the future as more exciting and interesting, thus, future events evoke more emotions and curiosity which lead us to make more extreme predictions of the valence of future events (i.e., future heuristic; see Van Boven and Ashworth, 2007; Herbert, 2010). In addition, people in general have a sense of being able to influence the future; therefore, most of us use narratives of the future to promote behavior that is beneficial for ourselves or our group. For example, the Martin Luther King Jr. “I have a dream” speech communicates a positively framed future with desirable values, such as, tolerance and justice. Importantly, the research reviewed here, suggest that the same should hold for negative events, that is, if we are imagining or speaking about a negative event that might happen in the future, we value it more negatively than if we imagine or speak about the same event as if it already have happened in the past (e.g., Caruso, 2010). However, we argue that this temporal asymmetry (i.e., future > past, or past < future, for both positive and negative events) needs to be tested using real life data (cf. Hsee et al., 2014), because in contrast to experimental designs, people typically talk, or write, about different topics and events when making statements about the past, the present, and the future. In other words, the occurrence of positive versus negative past/present/future events in everyday narratives differs from that of experimental controlled designs, which, for good reasons, always present and equal amount of positive and negative events.

These everyday life narratives of past, present, and future events are possible thanks to human beings’ unique ability to mental time travel (Suddendorf and Corballis, 1997). These narratives of positive and negative statements of future and past events might influence how humans perceive and recall emotional events. In this context, the ability to react fast to dangerous or negative stimuli is considered essential for an organism to ensure its survival. For example, in a series of experiments (Dijksterhuis and Aarts, 2003), participants detected negatively loaded words more accurately than positive ones, and this was true even when the words were presented subliminally, that is, so fast that the meaning of the words could not be explicitly understood. In other words, suggesting negative valence, rather than positive, as the most common state of being when humans imagine the past and the future. Indeed, a vast amount of research supports the notion that “bad is stronger than good” (Baumeister et al., 2001, p. 323). This includes findings showing that negative emotions, negative feedback, and negative major life events have greater impact in our physical, psychological and social health than positive ones. This underlying precedence of negativity is also reflected in our language: negative emotions have been shown to be overrepresented in the English language by approximately a 3/5 ratio and this ratio is even stronger (3/4) regarding words describing personality traits (for a review see Baumeister et al., 2001). On this basis, we could expect that an “I have a nightmare” speech would be the most common scenario when people imagine the future.

However, other empirical evidence emphasizes the importance and prevalence of positivity. For example, the analysis of the 5,000 most frequently used words in Twitter, lyrics, books, and the New York Times, suggested an overrepresentation of positive words (Dodds et al., 2011; Kloumann et al., 2012; see also Kramer et al., 2014 for research on emotional contagion in social networks). Moreover, when people imagine a future or past event, positive information is accessed more easily making it more central to the construction of the imagined event (D’Argembeau and Van der Linden, 2004). Perhaps because positive information is more contextual, leading to the construction of more positive and richer imagined future and past events. For instance, despite our tendency to detect negative stimuli faster, negative stimuli are more difficult to remember after longer delays compared to neutral and positive stimuli (Szpunar et al., 2012). That is, showing that humans have a fallacy for a “rose simulated future” (Szpunar, 2010; Szpunar et al., 2012; see also research on self-enhancement and positivity bias; D’Argembeau and Van der Linden, 2004).

This fallacy of a “rosy simulated future,” however, might as well be part of what makes people healthy. As the matter of fact, the apprehension of events is also related to peoples’ self-regulation (Higgins, 1997; see also Garcia et al., 2010). The “I have a dream” speech is a good example of promotion focused regulation, because it is based on envisioning a successful and bright future (cf. Higgins, 1997). In contrast, people might have a prevention focus when constructing and communicating future events; for example, by envisioning failure and being more vigilant about forthcoming events, in order to avoid or prevent such a dark future (cf. Higgins, 1997). Thus, promotion and prevention focus are important motivators of behavior and even mental health^2^ (Higgins, 1997; Amato et al., 2017; Garcia et al., 2017; Amato and Garcia, 2018; see also Walker et al., 2003; D’Argembeau and Van der Linden, 2004). From this perspective, speeches or narratives that envision the promotion of a brighter future or preventing a dark future; both communicate a pleasant or desired state because the individual either envisions a happy and pleasant future or the pleasant relief by avoiding dark or bad outcomes (cf. Higgins, 1997). People, for instance, strive to create legacies that will survive beyond their own existence (Wade-Benzoni and Tost, 2009). Accordingly, having the belief that one has made a difference and will leave the world a better place (cf. promotion focus) leads to the sense of purpose and meaning in life (Wade-Benzoni, 2003; de St Aubin et al., 2004; Grant and Wade-Benzoni, 2009). The motivation to not leave a negative legacy behind (cf. prevention focus) is of equal importance; imposing burdens on powerless others is morally problematic for us humans (Wade-Benzoni and Tost, 2009). Hence, in relation to mental time travel and both positive and negative events, an individual’s everyday narratives could be expected to both promote bright futures and prevent dark futures, in turn, devaluating the past.

### The Present Study

In summary, findings reviewed here on how we humans evaluate events when we use our ability to mental time travel are complex. First of all, positive events are evaluated as more positive and negative events are evaluated as more negative when these are imagined to happen in the future rather than have happened in the past. Secondly, even if we perceive negative stimuli faster, we selectively prefer to retrieve positive aspects of both past and future events. That being said, since we have a positive heuristic for the future (Herbert, 2010), valence of imagined/constructed future events should be expected to be higher and more positive than recalled/reconstructed past events. Last but not the least, self-regulation theory suggests that both promotion and prevention focus are used to regulate behavior toward desirable positive states (e.g., achieving a desired future or avoiding an undesired future). Hence, narratives and statements from real life, containing a mixture of positive and negative events, could be expected to reiterate a brighter (i.e., promotion focus statements) and less dark (i.e., prevention) future.

The examination of real-life data is important from a methodological perspective (Fischhoff, 1996). For instance, when people reconstruct the past, the present, and the future, the number of positive and negative events is not evenly distributed across temporal dimensions. Since current and predominant views in a society tend to perpetuate themselves through their recurrent presentation in the media (e.g., newspapers, social networks, popular songs) (Garcia and Sikström, 2013a; Garcia et al., 2016), we investigated the temporal valence asymmetry of events using two large text corpora from online newspapers and Facebook status updates by applying the LSA algorithm to quantify the valence of the words (see also Kjell et al., 2018). Specifically, as in previous research we were interested in the valence related to events placed in different temporal dimensions; but in contrast to past research, we did not compare the valence of identical hypothetical events occurring in the past or the future. Instead, we investigated the valence of any events that journalist and Facebook users choose to write about.

## Materials and Methods

### Ethics Statement

This research protocol was approved by the Ethics Committee of Lund University.

### Participants

The first data set comprised news stories from Reuters during 1997. We chose this corpus because it was one of the few large news corpus that were public available at the time when the research was conducted. In addition, a few thousand Facebook users also provided us with 1,183,180 status updates (see the myPersonality project^3^). The Facebook data was collected during 2009 through 2011.

### Statistical Method and Procedure

We quantified the valence of temporal markers (i.e., words representing the past, the present, and the future, respectively) using the LSA algorithm. The analyses were conducted in a web-based automated program for analyses of quantitative semantics called semanticexcel,^4^ which was developed by one of the authors of this paper. Technical details of how this software generates a semantic representation and predict numbers (valence) from a text based on this representation can be found elsewhere (see Roll et al., 2012, for predicting abstractness; Garcia and Sikström, 2013b, for predicting affectivity scores; and Garcia and Sikström, 2014; Garcia et al., 2015 for predicting personality scores; see also Kjell et al., 2018). Here we just present a brief overview.

Semanticexcel contains semantic representations of several languages, including English. The representation of English used here was generated for the 1997 Reuter news corpus. First, a matrix is generated were rows corresponds to unique single words and each column corresponds to context to the words in the corpus. The rows consisted of the 120,000 most frequency words in corpora, whereas the columns consisted of the contexts of the 10,000 most common words. The contexts of the words were generated from the fifteen words preceding, and fifteen words following, the word in each column. Thus, cells in this matrix represent the frequency of occurrence of a word (rows) within a context of a word (columns). For example, the word “grateful” may have a frequency *f_1_* in the context “aiding” and a frequency *f_2_* in the context “accidents.” In this way, every word is represented by an array of frequencies of occurrence in each related context to a word.

A basic assumption is that words with similar meaning tend to occur in the same contexts. This implies that the vectors representing similar words should point in similar direction (Sun, 2008). However, to get a good semantic representation this word-by-context sample matrix needs to be compressed to a smaller word-by-semantic dimension matrix, where this smaller matrix tends to create a more generalized semantic representation. We conducted this data compression using Singular Value Decomposition (Strang, 1998), a widespread dimensionality-reduction technique similar to Principal Component Analysis. The resulting matrix is called a semantic space, which describes the semantic relatedness between words. This method has a high level of accuracy, comparable to human performance in different tasks, such as, rating grades (e.g., Landauer and Dumais, 1997, Landauer et al., 1998, Howard and Kahana, 2002). In our analysis, the resulting semantic representation consisted of 120,000 words, where each word is represented in a vector consisting of 100 dimensions.

These representations were used to predict/estimate the valence of each article/status update, respectively, in relation to the time markers (years and verbs were selected as time markers). In the present study, we first identified words related to the past, the present, and the future (i.e., target words). Then we evaluate, using LSA, whether the contexts (the context is defined as the 15 words preceding or following each target word) these words were written in consist of positive or negative words (i.e., the valence). For the sake of clarity, we first briefly describe the rationale behind the chosen time markers, then how we computed valence and then how we did the statistical analyses for testing our hypotheses.

Year-data were divided into categories relative to the publication date. In the Reuter news corpus, the year condition of groups was arranged around the year 1997. By comparing the year that the articles were written, which in the Reuter data was 1997, we identified 1994–1996, as markers of the past, whereas 1998 and 1999, as makers for the future. In the Facebook corpus, this was based on the context content in relation to when the users’ status was published. For target words in both Reuter and Facebook data, the verbs were chosen by randomly selecting verbs from McMillan’s essential dictionary (Rundell and Fox, 2003). Random selection was used to minimize author bias. This method generated a list of 10 solid past conjugations (see **Table 1**). The English language lack unambiguous usage of the future tense; auxiliary verbs (i.e., verbs that add functional or grammatical meaning and usually accompany a main verb in infinitive) are often needed to imply future tense (Leech, 2004). Some conjugations can be used to describe past, present and/or future (e.g., “Fall” can be used in multiple ways: I Fall [present] and I will Fall [future]). To analyze the future tense, we therefore relied on the fact that this is a modal construction which uses auxiliaries (will or shall) + infinitive (Leech, 2004). Hence, only these two auxiliaries (“will” and “shall”) without the infinitive were analyzed to represent the future tense, with the assumption that these are the most frequently used auxiliaries to imply future tense. It should be noted that these auxiliaries can refer to events in the near or far away future, which implies that our data is likely to contain referrals to both near and far away future events. These auxiliaries are shown in **Table 1**.

**Table 1:** Verbs and auxiliaries analyzed.

Infinitive	Simple Past (Past)	Past Participle (Present)	Auxiliaries (Future)
Fall	Fell^∗^	Fallen^∗^	Will^∗^
Go	Went^∗^	Gone^∗^	Shall^∗^
Grow	Grew^∗^	Grown^∗^	
Speak	Spoke^∗^	Spoken^∗^	
Be	Was^∗^	Been^∗^	
Write	Wrote^∗^	Written^∗^	
Eat	Ate^∗^	Eaten^∗^	
Drive	Drove^∗^	Driven^∗^	
Do	Did^∗^	Done^∗^	
Choose	Chose^∗^	Chosen^∗^	


*^∗^ = Words analyzed in the “Verb” condition.*

The method used for predicting the valence of words was multiple-linear regression (y = c^∗^x), where the semantic representations (x) is used as predictors, which are trained on a limited number of words ranked by valence (y). The ANEW (Affective Norms for English Words) wordlist, (Bradley and Lang, 1999) was used to identify one thousand words ranked on valence. Multiple-linear regression was performed between the ANEW list and the semantic space. The resulting regression coefficients (c) can then be used to predict the valence of all words represented in the semantic space. The validity of this method, was estimated with a leave-one-out procedure so that the tested word was removed from the training set, showing a high correlation between predicted and rated scores (*r* = 0.62). Thus, the LSA algorithm generalizes from the evaluation of a small set of ANEW words, to all words in the semantic representation, and thus allows estimation of the valence of a larger number of words, compared to simply counting and affective score based on their ANEW values. We calculated the average valence for words in contexts for target words. This provides a more reliable means of measuring valance; where every single context of a target word has an average predicted valence, rather than the estimated valence of just a target word. In both corpora, 10,000 articles were scanned to obtain the valence of the contexts.

A One-way Analysis of Variance (ANOVA) was computed for each variable in both corpora. In each analysis three conditions were created (Past, Present, and Future). The verbs were assigned into the Past condition if it was written in Simple Past and the Present condition if it was written in Past Participle. Auxiliaries were assigned into the Future condition. Years were assigned to the Past condition if written earlier than the publication date(s), to the Present condition if they were the publication date(s), and the Past condition if written later than the publication date(s). In both corpora, post-hoc two-tailed independent *t*-tests were conducted to examine the difference in valence between the Past and the Present, and the Present and the Future.

## Results

### Verbs (Reuters)

**FIGURE 1:**
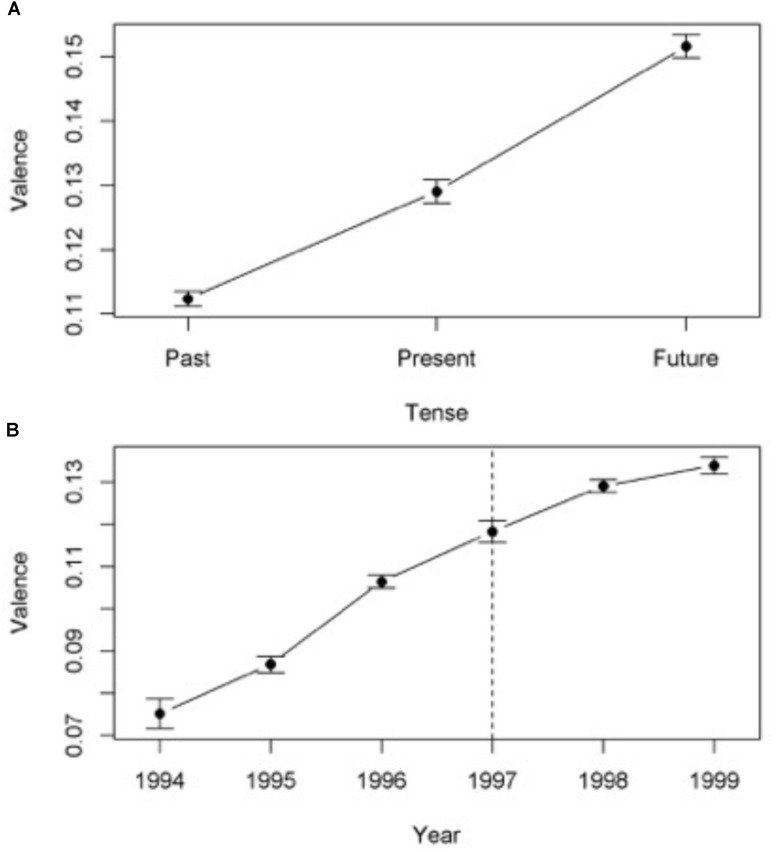
**(A)** Mean Valence and confidence intervals for Reuter Verbs in Past, Present, and Future tense. **(B)** Mean Valence and confidence intervals for Reuter Years. Dotted line marks the relative publication date.

Ten verbs and two auxiliaries from 10,000 documents produced 14,165 contexts, where some documents produced more than one context. The mean and standard deviation for the valence associated to each group is presented in **Figure 1A**. The frequency of occurrence of each verb and condition can be found in **Table 2A**. We conducted an ANOVA to investigate if the valence of the contexts differed between the three conditions: Past, Present and Future [*F* = 164.0, *df* = 2, 14162, *p* < 0.001, η*^2^* = 0.023, 95% *CI*(0.018, 0.027)]. Homogeneity of variances was significant at the 0.001 level (*Levene* = 13.17, *df* = 2, 14162). A two-tailed independent sample *t-test* showed that there was a significant difference between Past and Present [*t*(11181) = -7.95, *p* < 0.001, *d* = -0.162, 95% *CI*(-0.201, -121)] and between Present and Future [*t*(6504) = -8.98, *p* < 0.001, *d* = 0.223, 95% *CI*(-0.272, -0.174)]. In other words, the Present had higher valence than the Past, while the Future had higher valence than the Present. The effects sizes were, however, weak.

**Table 2A:** Verb frequency, proportions of verbs and proportions of conditions in the Reuter corpus.

Condition	Verb	Frequency	Verb proportions relative to corpus size	Condition proportions relative to corpus size
Future	Shall	992	7.00%	
Future	Will	1990	14.05%	21.05%
Past	Ate	222	1.57%	
Past	Chose	495	3.49%	
Past	Did	930	6.57%	
Past	Drove	446	3.15%	
Past	Fell	1240	8.75%	
Past	Grew	824	5.82%	
Past	Spoke	980	6.92%	
Past	Was	1080	7.62%	
Past	Went	482	3.40%	
Past	Wrote	720	5.08%	52.38%
Present	Been	278	1.96%	
Present	Chosen	743	5.25%	
Present	Done	240	1.69%	
Present	Driven	595	4.20%	
Present	Eaten	169	1.19%	
Present	Fallen	124	0.88%	
Present	Gone	194	1.37%	
Present	Grown	334	2.36%	
Present	Spoken	317	2.24%	
Present	Written	770	5.44%	26.57%
Total		14165	100,00%	100,00%


### Years (Reuters)

Data from six years was analyzed, generating a total of 16,396 contexts.

The mean and standard deviation of the valence for each group can be found in **Figure 1B**. The frequency of occurrence of each year and condition can be found in **Table 2B**. An ANOVA reveled a significant difference in valence between the groups [*F* = 114.22, *df* = 5, 16390, *p* < 0.001, η*^2^* = 0.038, 90% *CI*(0.029, 0.039)]. Homogeneity of variances was significant at the 0.001 level (*Levene* = 13.238, *df* = 5, 16390). A two-tailed independent sample *t-test* showed that there was a significant difference in valence between Past and Present [*t*(9520) = -7.99, *p* < 0.001, *d* = -0.221, 95% *CI*(-0.275, -0.166)] and between Present and Future [*t*(8438) = -4.55, *p* < 0.001, *d* = -0.130, 95% *CI*(-0.182, -0.072)]. In other words, as for the verbs, the Present had higher valence than the Past, while the Future had higher valence than the Present. The effects sizes were, however, weak.

**Table 2B:** Year frequency, proportions of years and proportions of conditions in the Reuter corpus.

Condition	Year	Frequency	Year proportions relative to corpus size	Condition proportions relative to corpus size
Future	1998	3996	24.37%	
Future	1999	2878	17.55%	41.92%
Past	1996	4156	25.35%	
Past	1995	2861	17.45%	
Past	1994	939	5.73%	48.52%
Present	1997	1566	9.55%	9.55%
Total		16396	100.00%	100.00%


### Verbs (Facebook)

**FIGURE 2:**
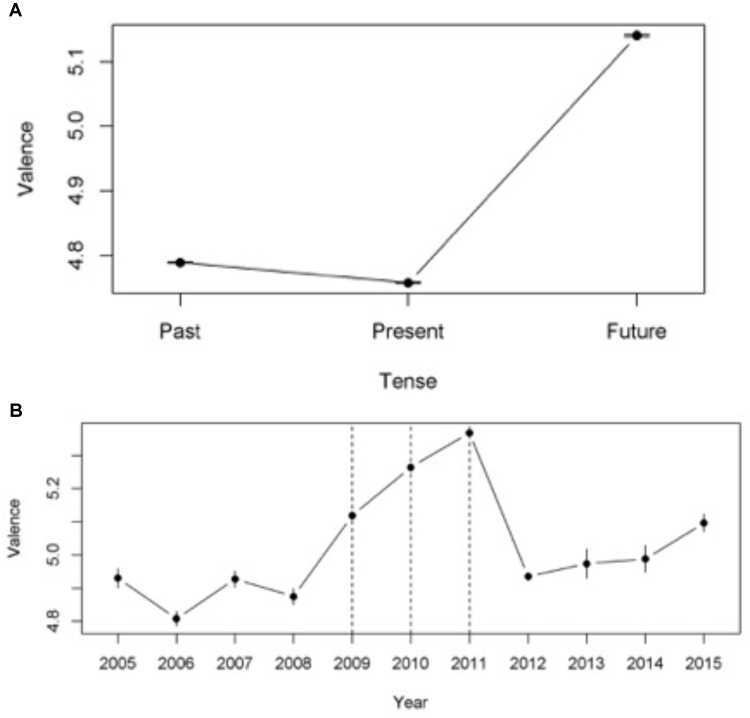
**(A)** Mean Valence and confidence intervals for Facebook Verbs in Past, Present, and Future tense. **(B)** Mean Valence and confidence intervals for Facebook Years. Dotted line marks the relative publication dates.

Ten verbs and two auxiliaries from 10,000 documents produced 860,127 contexts, where some documents produced more than one context. The mean and standard deviation for the valence associated to each group is presented in **Figure 2A**. The frequency of occurrence of each verb and condition can be found in **Table 3A**. An ANOVA reveled a significant difference in valence between the groups [*F* = 16717, *df* = 2, 858668, *p* < 0.001, η*^2^* = 0.038, 90% *CI*(0.037, 0.038)]. Homogeneity of variances was significant at the 0.001 level (*Levene* = 371.55, *df* = 2,858668). A two-tailed independent sample *t-test* showed that there was a significant difference in valence between Past and Present [*t*(716660) = 18.98, *p* < 0.001, *d* = -0.45, 95% *CI*(-0.050, -0.041)] and between Present and Future [*t*(458000) = -172.71, *p* < 0.001, *d* = 0.55, 95% *CI*(0.546, 0.558)]. In other words, conversely to findings in the Reuters data, the Present had lower valence than the Past. However, in line with Reuters’ findings, the Future had higher valence than the Present. The effects sizes were weak or close to moderate.

**Table 3A:** Verb frequency, proportions of verbs and proportions of conditions in the Facebook corpus.

Condition	Verb	Frequency	Verb proportions relative to corpus size	Condition proportions relative to corpus size
Future	Shall	42011	4.88%	
Future	Will	100000	11.63%	16.51%
Past	Ate	28653	3.33%	
Past	Chose	6440	0.75%	
Past	Did	100000	11.63%	
Past	Drove	10421	1.21%	
Past	Fell	30519	3.55%	
Past	Grew	6656	0.77%	
Past	Spoke	6423	0.75%	
Past	Was	100000	11.63%	
Past	Went	100000	11.63%	
Past	Wrote	13014	1.51%	46.75%
Present	Been	100000	11.63%	
Present	Chosen	3639	0.42%	
Present	Done	100000	11.63%	
Present	Driven	2500	0.29%	
Present	Eaten	8919	1.04%	
Present	Fallen	8875	1.03%	
Present	Gone	66040	7.68%	
Present	Grown	12203	1.42%	
Present	Spoken	3150	0.37%	
Present	Written	10664	1.24%	36.74%
Total		860127	100.00%	100.00%


### Years (Facebook)

Data from eleven years were analyzed generating a total of 64,009 contexts. The mean and standard deviation of the valence for each group can be found in **Figure 2B**. The frequency of occurrence of each verb and condition can be found in **Table 3B**. An ANOVA reveled a significant difference between the groups [*F* = 182.2, *df* = 10, 63513, *p* < 0.001, η*^2^* = 0.028, 90% *CI*(0.026, 0.030)]. Homogeneity of variances was significant at the 0.001 level (*Levene* = 96.09, *df* = 2, 63521). A two-tailed independent sample *t-test* showed that there was a significant difference in valence between Past and Present [*t*(58751) = -27.86, *p* < 0.001, *d* = 0.48, 95% *CI*(0.446, 0.513)] and between Present and Future [*t*(59925) = 29.05, *p* < 0.001, *d* = 0.10, 95% *CI*(0.06, 0.146)]. In other words, as for the Reuters’ findings, Present had higher valence than the Past, while the Future had higher valence than the Present. The effects sizes were, however, weak.

**Table 3B:** Year frequency, proportions of years and proportions of conditions in the Facebook corpus.

Condition	Year	Frequency	Year proportions relative to corpus size	Condition proportions relative to corpus size
Future	2012	3753	5.86%	
Future	2013	324	0.51%	
Future	2014	327	0.51%	
Future	2015	398	0.62%	7.50%
Past	2005	645	1.01%	
Past	2006	1016	1.59%	
Past	2007	951	1.49%	
Past	2008	1069	1.67%	5.75%
Present	2009	4759	7.43%	
Present	2010	28515	44.55%	
Present	2011	22252	34.76%	86.75%
Total		64009	100.00%	100.00%


## Discussion

We investigated the temporal valence asymmetry of events using real-life data (i.e., two large text corpora from online newspapers and Facebook status updates) by applying language processing methods and tools. We identified specific words or target words in the narratives at hand in relations to time markers of the past, the present and the future. We then measured the valence of the contexts (the context is defined as the 15 words preceding or following each target word) in which these target words appeared. Our results using verbs as temporal markers showed, in both the Reuter and Facebook corpus, that valence for the future was significantly higher (i.e., more positive) compared to the past (future > past). Similarly, in the Reuter year condition, valence increased approximately linear from 1994 to 1999. In the Facebook year condition, it is also evident that the valence of the future is significantly higher (i.e., more positive) than past valence. However, for the Facebook data, 2012 did not differ significantly in valence compared to 2007. Nevertheless, the analyses of the Reuters data indicated that the past is devaluated against both the present and the future, while the analyses of the Facebook data indicated that both the past and the present are devaluated against the future. That is, by either devaluating the past against the future or by devaluating the present against the future, both people who engage in the “I have a dream” speech or the “I have a nightmare” speech try always to reach a more pleasant state (cf. Higgins, 1997).

In the present study, the future seems to be valued positively higher than the past, even though current research suggest that evaluations of the future should be more extreme both when it comes to negative and positive events (Caruso et al., 2008; Caruso, 2010). This is even more accentuated in the Reuters data set, which is striking, considering that there was a high likelihood that the sample would include an overrepresentation of lower valence contexts. For instance, news stories that have a more negative valence are twice as likely to be featured in print (Soroka, 2012; see also Trussler and Soroka, 2014). According to the future heuristic, the future is more exciting and interesting, thus, evoking more emotions and curiosity (Herbert, 2010). However, this heuristic only explains that more emotions, both positive and negative, should be associated to texts found in the context of future time-markers. That is, the future heuristic only explains the temporal asymmetry (i.e., past vs. future), not the valence asymmetry found in the present study. Our results, however, might mirror our increased excitement about the future compared to the past (i.e., the future heuristic) in conjunction with our tendency to favor positive information when imagining future events (i.e., positivity bias). This positive excitement about the future is probably based on a solid foundation derived from our concrete perception and physical interaction with the world (i.e., cognitive scaffolding; Herbert, 2010). We humans move forward, and not backward, which in turn might explain why concepts like “progress” and “advancement” are generally associated to something good, while “backward thinking” is often regarded as bad (see Herbert, 2010, for more examples such as “up vs. down”). Indeed, people seek to make a positive impression upon the world by leaving a legacy that will transcend themselves into future generations (e.g., Wade-Benzoni, 2003; de St Aubin et al., 2004; Grant and Wade-Benzoni, 2009; Wade-Benzoni et al., 2010).

### Strengths and Limitations

The quantification of language by extracting words from contexts is a powerful research tool when a large amount of data is available (Landauer and Dumais, 1997; Landauer et al., 1998; Howard and Kahana, 2002; Arvidsson et al., 2011). That being said, research using similar methods in social psychology is limited, making it difficult to compare our findings with previous research. To the best of our knowledge, no previous studies have used the proposed method to examine how people’s ability to time travel influences how they evaluate events or rather how it influences the valence in their narratives. One of the strengths of the present study is that we analyzed data from two different domains and found the same overall pattern, that is, that the past is devaluated compared to the future. However, the effect sizes were between weak to moderate. Thus, further experimental and empirical data is needed to confirm or disprove our findings. For instance, it is plausible that narratives of events by non-journalists might give different results. Quoidbach (2013), for example, suggested that there are differences between the cognitive processes that allow people to look forward and backward in time—imagining new things is generally more difficult than reconstructing old ones from one’s personal life. These researchers suggest that, because people find it difficult to imagine themselves changing in the future (e.g., their personality, preferences), people think that it is unlikely the they will actually change (see also Gärling and Gamble, 2012; Garcia et al., 2014). In other words, if people in general find it difficult to change, it is possible that the future is as “rosy” as both the past and the present. In that case, the news and social media data presented here is only a reflection of a contagion of positive emotions for events placed in the future.

Moreover, auxiliary verbs (i.e., verbs that add functional or grammatical meaning and usually accompany a main verb in infinitive) sometimes have other meanings, than implying future tense. For example, “will” or “shall” can in conversational language be used in the present tense to express an ongoing activity that continuous in the near future. Although such exceptions may exist, the most common usage of “will” or “shall” is to describe future events or activities. Common for all verbs, that we used as temporal markers, is also that they are typically used within their denoted tense. Another limitation of the study is that predicting valence using the LSA method may introduce errors in the calculation. Although this is true, we still believe that the LSA is a powerful method that allows automatic measuring of valence with reasonable good accuracy.

Finally, we acknowledge the uneven proportions of extracted verbs and years in the Past, Present and Future conditions. At the most extreme, the years from the Facebook corpus was skewed in the sense that almost 87% of the extracted data was assigned to the Present condition. Most of the data showed the same type of skewness. The verbs from both data sets being the least skewed.

### Further Research and Concluding Remarks

Our results open up a number of questions for future research. First, the choice of temporal markers can be further elaborated. Here we chose the time markers based on which words are commonly used as temporal markers in everyday language. Secondly, it would be interesting to replicate the results using different text corpora, such as, literature, novels and short stories, and political speeches. Moreover, there might be cultural differences in how we perceive and represent the past, the present, and the future. For instance, Chinese people seem to recall events from the past in greater detail compared to Canadians (Ji et al., 2009). Also in this line, one’s worldview or conception of the world might influence our preference for past or future mental time travel (Ettlin and Hertwig, 2012).

All this being said, our results suggest that the evaluative communication of an event is temporal-valence asymmetrical (that is, valence of an event in time = past < present < future). The outcome, however, depends on whether it can function as incitement for future action or the promotion of behavior (higher valence) or feedback from past actions to avoid or prevent behavior in the future (lower valence): The Time Anchored Incitement Hypothesis (TAIH). We argue that, it might be self-beneficial to the one being the speaker to convey positive evaluative statements about the future that are in line with the legacy she/he envisions to leave for future generations, which in turn also makes the speaker to appear as more appealing and exciting to listeners. After all, we seem to have bias toward a “rosy future.” On the other hand, the negative value associated to past events might signal both danger and its proximity (Kyung et al., 2010), thus, focusing attention on improving or even avoiding past behaviors.

## Author Contributions

DG and CA wrote the paper and revised drafts of the paper. KD and SS conceived and designed the experiments, performed the experiments, analyzed the data, contributed reagents, materials, and analysis tools, wrote the paper, prepared figures and/or tables, and reviewed drafts of the paper. MK, KD, SS, and CA reviewed drafts of the paper.

## Conflict of Interest Statement

The authors declare that the research was conducted in the absence of any commercial or financial relationships that could be construed as a potential conflict of interest.

## Footnotes

1 Here we use the term high valence as positive and low valence as negative.

2 Even if both types of regulatory focus motivate individuals toward attention to future states (i.e., a brighter future and a less dark future), people’s behavior (e.g., action, inaction, counteraction) might differ depending on which type of future is being envisioned.

3 https://sites.google.com/michalkosinski.com/mypersonality

4 www.semanticexcel.com

## References

[B1] Amato C. Garcia D. (2018). “Regulatory Mode,” in *Encyclopedia of Personality and Individual Differences*, eds Zeigler-Hill V. Shackelford T. (Cham: Springer), 1–9. 10.1007/978-3-319-28099-8_2305-1

[B2] Amato C. Nima A. A. Mihailovic M. Garcia D. (2017). Modus operandi and affect in sweden: the swedish version of the regulatory mode questionnaire. *PeerJ* 5:e4092. 10.7717/peerj.4092 29181282 PMC5702250

[B3] Arvidsson D. Werbart A. Sikström S. (2011). Changes in object representations measured by a semantic space method. *Psychother. Res.* 21 430–446. 10.1080/10503307.2011.577824 21623547

[B4] Baumeister R. F. Bratslavsky E. Finkenauer C. Vohs K. D. (2001). Bad is stronger than good. *Rev. Gen. Psychol.* 5:323 10.1037/1089-2680.5.4.323

[B5] Bradley M. Lang P. (1999). *Affective Norms for English Words (ANEW): Instruction Manual and Affective Ratings.* Gainesville, FL: University of Florida.

[B6] Caruso M. (2010). When the future feels worse than the past: a temporal inconsistency in moral judgment. *J. Exp. Psychol. Gen.* 139 610–624. 10.1037/a0020757 20853992

[B7] Caruso M. Gilbert T. Wilson T. (2008). A wrinkle in time. *Psychol. Sci.* 19 796–801. 10.1111/j.1467-9280.2008.02159.x 18816287

[B8] D’Argembeau A. Van der Linden M. (2004). Phenomenal characteristics associated with projecting oneself back into the past and forward into the future: influence of valence and temporal distance. *Cons. Cogn.* 13 844–858. 10.1016/j.concog.2004.07.007 15522635

[B9] de St Aubin E. McAdams D. P. Kim T. (2004). *The Generative Society: Caring for Future Generations.* Washington, DC: American Psychological Association 10.1037/10622-000

[B10] Dijksterhuis A. Aarts H. (2003). On wildebeests and humans: The preferential detection of negative stimuli. *Psychol. Sci.* 14 14–18. 10.1111/1467-9280.t01-1-01412 12564748

[B11] Dodds P. S. Harris K. D. Kloumann I. M. Bliss C. A. Danforth C. M. (2011). Temporal patterns of happiness and information in a global social network: hedonometrics and twitter. *PLoS One* 6:e26752. 10.1371/journal.pone.0026752arXiv:1101.5120v5 22163266 PMC3233600

[B12] Landauer T. Dumais S. (1997). A solution to plato’s problem: the latent semantic analysis theory of acquisition, induction, and representation of knowledge. *Psychol. Rev.* 104 211–240. 10.1037/0033-295X.104.2.211

[B13] Ettlin F. Hertwig R. (2012). Back or to the future? Preferences of time travelers. *Judge. Decis. Mak.* 7 373–382.

[B14] Fischhoff B. (1996). The real world: what good is it? *Organ. Behav. Hum. Decis. Processes* 65 232–248. 10.1006/obhd.1996.0024

[B15] Garcia D. Anckarsäter H. Kjell O. N. E. Archer T. Rosenberg P. Cloninger C. R. (2015). Agentic, communal, and spiritual traits are related to the semantic representation of written narratives of positive and negative life events. *Psychol. Well Being Theory Res. Pract.* 5 1–20. 10.1186/s13612-015-0035-x

[B16] Garcia D. Ghiabi B. Nima A. A. Archer T. (2014). The end of happiness: temporal distance and judgments of life satisfaction in Sweden, Iran, Spain, and El Salvador. *Int. J. Happiness Dev.* 2 371–382. 10.1504/IJHD.2015.073945

[B17] Garcia D. Kjell O. N. E. Sikström S. (2016). “A Collective Picture of What Makes People Happy: Words Representing Social Relationships, not Money or Material Things, are Recurrent with the Word ‘Happiness’ in Online Newspapers,” in *The Psychology of Social Networking. Identity and Relationships in Online Communities* Vol. 2 eds Riva G. Wiederhold B. K. Cipresso P. (Berlin: DeGruyter Open).

[B18] Garcia D. Rosenberg P. Erlandsson A. Siddiqui A. (2010). On lions and adolescents: affective temperaments and the influence of negative stimuli on memory. *J. Happiness Stud.* 11 477–495. 10.1007/s10902-009-9153-6

[B19] Garcia D. Rosenberg P. Lindskär E. Amato C. Nima A. A. (2017). The swedish version of the regulatory mode questionnaire. *Data Brief* 14 251–254. 10.1016/j.dib.2017.07.050 28795102 PMC5540705

[B20] Garcia D. Sikström S. (2013a). A collective theory of happiness: words related to the word happiness in swedish online newspapers. *Cyberpsychol. Behav. Soc. Netw.* 16 469–472. 10.1089/cyber.2012.0535 23621718

[B21] Garcia D. Sikström S. (2013b). Quantifying the semantic representations of adolescents’ memories of positive and negative life events. *J. Happiness Stud.* 2012. 14 1309–1323. 10.1007/s10902-012-9385-8

[B22] Garcia D. Sikström S. (2014). The Dark Side of Facebook – dark triad of personality predicts semantic representation of status updates. *Pers. Individ. Diff.* 67 92–94. 10.1016/j.paid.2013.10.001

[B23] Gärling T. Gamble A. (2012). Influences on current mood of eliciting life-satisfaction judgments. *J. Posit. Psychol.* 7 219–229. 10.1080/17439760.2012.674547

[B24] Grant A. M. Wade-Benzoni K. A. (2009). The hot and cool of death awareness at work: mortality cues, aging, and self-protective and prosocial motivations. *Acad. Manag. Rev.* 34 600–622.

[B25] Herbert W. (2010). *On Second Thought: Outsmarting Your Mind’s Hard-Wired Habits.* New York, NY: Crown Publishers.

[B26] Higgins E. T. (1997). Beyond pleasure and pain. *Am. Psychol.* 52 1280–1300. 10.1037/0003-066X.52.12.1280 9414606

[B27] Howard M. Kahana M. (2002). A distributed representation of temporal context. *J. Math. Psychol.* 46 269–299. 10.1006/jmps.2001.1388

[B28] Hsee C. K. Rottenstreich Y. Tang J. (2014). Asymmetries between positives and negatives. *Soc. Personal. Psychol. Compass* 8 699–707. 10.1111/spc3.12143 18540960

[B29] Ji L. J. Guo T. Zhang Z. Messervey D. (2009). Looking into the past: cultural differences in perception and representation of past information. *J. Pers. Soc. Psychol.* 96 761–769. 10.1037/a0014498 19309200

[B30] Kjell O. N. E. Kjell K. Garcia D. Sikström S. (2018). Semantic measures: using natural language processing to measure, differentiate and describe psychological constructs. *Psychol. Methods* 10.1037/met0000191 [Epub ahead of print]. 29963879

[B31] Kloumann I. M. Danforth C. M. Harris K. D. Bliss C. A. Dodds P. S. (2012). Positivity of the english language. *PLoS One* 7:e29484. 10.1371/journal.pone.0029484 22247779 PMC3256157

[B32] Kramer A. D. Guillory J. E. Hancock J. T. (2014). Experimental evidence of massive-scale emotional contagion through social networks. *Proc. Natl. Acad. Sci. U.S.A.* 7:e29484. 10.1371/journal.pone.0029484 24889601 PMC4066473

[B33] Kurtz J. (2008). Looking to the future to appreciate the present. the benefits of perceived temporal scarcity. *Psychol. Sci.* 19 1238–1241. 10.1111/j.1467-9280.2008.02231.x 19121130

[B34] Kyung E. J. Menon G. Trope Y. (2010). Reconstruction of things past: why do some memories feel so close and others so far away? *J. Exp. Soc. Psychol.* 46 217–220. 10.1016/j.jesp.2009.09.003 21836727 PMC3152818

[B35] Landauer T. K. Foltz P. W. Laham D. (1998). An introduction to latent semantic analysis. *Discourse Process.* 25 259–284. 10.1080/01638539809545028

[B36] Leech G. (2004). *Meaning of the English Verb.* Edinburgh: Pearson Education Limited.

[B37] Quoidbach J. Gilbert D. T. Wilson T. D. (2013). The end of history illusion. *Science* 339 96–98. 10.1126/science.1229294 23288539

[B38] Roll M. Mårtensson F. Sikström S. Apt P. Arnling-Bååth R. Horne M. (2012). Atypical associations to abstract words in Broca’s aphasia. *Cortex* 48 1068–1072. 10.1016/j.cortex.2011.11.009 22172978

[B39] Rundell M. Fox G. (2003). *Macmillans Essential Dictionary for Intermediate Learners.* London: Macmillan Education.

[B40] Sikström S. (n.d.). *LSALAB.* Retrieved 3rd January 2011 from University of Lund. http://www.lucs.lu.se/sverker.sikstrom/LSALAB_intro.html

[B41] Soroka S. N. (2012). The gatekeeping function: distributions of information in media and the real world. *J. Polit.* 74 514–528. 10.1017/S002238161100171X

[B42] Strang G. (1998). *Introduction to Linear Algebra*, 3rd Edn. Wellesley, MA: Wellesley-Cambridge Press.

[B43] Suddendorf T. Corballis M. C. (1997). Mental time travel and the evolution of the human mind. *Genet. Soc. Gen. Psychol. Monogr.* 123 133–167. 9204544

[B44] Sun R. (2008). *The Cambridge Handbook of Computational Psychology.* Cambridge: Cambridge University Press 10.1017/CBO9780511816772

[B45] Szpunar K. K. (2010). Episodic future thought: an emerging concept. *Perspect. Psychol. Sci.* 5 142–162. 10.1177/1745691610362350 26162121

[B46] Szpunar K. K. Addis D. R. Schacter D.-L. (2012). Memory for emotional simulations: remembering a rosy future. *Psychol. Sci.* 23 24–29. 10.1177/0956797611422237 22138157 PMC3847671

[B47] Trope Y. Liberman N. (2000). Temporal construal and time-dependent changes in preference. *J. Pers. Soc. Psychol.* 79 876–889. 10.1037/0022-3514.79.6.876 11138758

[B48] Trussler M. Soroka S. (2014). Consumer demand for cynical and negative news frames. *Int. J. Press* 19 360–379. 10.1177/1940161214524832

[B49] Van Boven L. Ashworth L. (2007). Looking forward, looking back: anticipation is more evocative than retrospection. *J. Exp. Psychol.* 136 289–300. 10.1037/0096-3445.136.2.289 17500652

[B50] Wade-Benzoni K. A. (2003). “Intergenerational identification and cooperation in organizations and society,” in *Research on Managing Groups and Teams* Vol. 5 eds Neale M. Mannix E. Polzer J. (Stamford, CT: JAI Press),257–277.

[B51] Wade-Benzoni K. A. Tost L. P. (2009). The egoism and altruism of intergenerational behavior. *Pers. Soc. Psychol. Rev.* 13 165–193. 10.1177/1088868309339317 19571118

[B52] Wade-Benzoni K. A. Sondak H. Galinsky A. D. (2010). Leaving a legacy: intergenerational allocations of benefits and burdens. *Business Ethics Q.* 20 7–34. 10.5840/beq20102013

[B53] Walker W. R. Skowronski J. J. Thompson C. P. (2003). Life is pleasant–and memory helps to keep it that way! *Rev. Gen. Psychol.* 7 203–210. 10.1037/1089-2680.7.2.203

[B54] Wilson R. E. Gosling S. D. Graham L. T. (2012). A review of facebook research in the social sciences. *Perspect. Psychol. Sci.* 7 203–220. 10.1177/1745691612442904 26168459

